# Blood type O increased perioperative blood loss in total knee arthroplasty: a propensity score-based inverse probability of treatment weighting matched analysis

**DOI:** 10.1186/s43019-025-00284-3

**Published:** 2025-07-25

**Authors:** Yoshihito Suda, Naoki Nakano, Yoshinori Takashima, Masanori Tsubosaka, Tomoyuki Kamenaga, Yuichi Kuroda, Kazunari Ishida, Shinya Hayashi, Ryosuke Kuroda, Tomoyuki Matsumoto

**Affiliations:** 1 Department of Orthopaedic Surgery, Kobe University Graduate School of Medicine, 7-5-1, Kusunoki-Cho, Chuo-Ku, Kobe, 650-0017 Japan; 2 Department of Orthopaedic Surgery, Kobe Kaisei Hospital, 3-11-15 Shinoharakita-Cho, Nada-Ku, Kobe, 657-0068 Japan

**Keywords:** ABO blood type, Bleeding, Total knee arthroplasty, Von Willebrand factor

## Abstract

**Purpose:**

The aim of this study was to compare total perioperative blood loss in patients undergoing total knee arthroplasty (TKA) in different ABO blood types.

**Methods:**

This study was approved by the Institutional Review Board of our hospital (IRB No. 1510) on 2 February 2015. A total of 260 knees undergoing unilateral primary cemented cruciate-retaining TKA for knee osteoarthritis were retrospectively registered. Perioperative total blood loss was calculated for each blood types based on the change in hematocrit from pre- to postoperative day 7, preoperative height and weight. Differences in blood loss among the four ABO blood types were assessed. The association between blood type (O versus others) and total blood loss was further evaluated after adjusting for baseline characteristics through inverse probability of treatment weighting (IPTW).

**Results:**

The study population was divided based on the ABO blood type: type O, 87; type A, 82; type B, 54; and type AB, 37. The mean total blood loss was 829 ± 370 ml for type O, 702 ± 384 ml for type A, 574 ± 361 ml for type B, and 528 ± 282 ml for type AB. Patients with blood Type O had significantly higher blood loss than all other blood types. After IPTW adjustment, mean total blood loss remained higher in type O (790 ± 370 ml) compared to other blood types (613 ± 364 ml).

**Conclusion:**

These findings suggest that blood type O may be associated with increased perioperative blood loss in TKA.

## Introduction

Proper control of blood loss during total knee arthroplasty (TKA) is essential [1]. Excessive bleeding during or after surgery may result in complications, including anemia, the need for transfusions, and an extended hospital stay [2, 3]. Various methods are currently used to manage blood loss during TKA, including postoperative flexion position [4], tranexamic acid [5, 6], cell salvage [7], and tourniquet [8, 9]. In TKA, total blood loss is made up of visible blood loss from the surgical site and wound drainage and hidden blood loss into the tissues. Previous report showed that the hidden blood loss is almost 50% of the total blood loss [10].

The ABO blood type system is defined by specific carbohydrate antigens that are found on various human cells, including red blood cells, platelets, and the vascular endothelium [11]. Recent studies have implicated that the ABO blood type is a potential risk of various diseases such as cancer [12], cardiocerebrovascular disease [13], hepatitis B virus infection [14], and venous thromboembolism [15]. Furthermore, various clinical conditions, including obstetric bleeding, upper gastrointestinal hemorrhage, and spontaneous intracerebral bleeding, have demonstrated an association between blood type and bleeding risk [16, 17]. Individuals with blood type O have been reported to have lower circulating levels of von Willebrand factor (vWF), a key glycoprotein in primary hemostasis, which may predispose them to increased bleeding risk. Although several studies have examined the influence of blood type on bleeding tendencies in other surgical fields, limited evidence exists regarding this association in orthopedic surgery, and to our knowledge, no previous study has specifically investigated this issue in the context of total knee arthroplasty. Our study builds upon this background by focusing on primary cemented TKA—a standardized procedure with relatively low surgical variability—and by using inverse probability of treatment weighting (IPTW) to minimize confounding between blood type groups. We hypothesized that patients with blood type O would experience greater blood loss during TKA than patients with other blood types. The purpose of this study was to evaluate the relationship between differences in ABO blood types and perioperative blood loss in patients undergoing TKA.

## Patients/methods

### Study design

Patients who received primary total knee arthroplasty at a single institution from April 2016 to September 2020 were retrospectively analyzed in this cohort study. The study protocol received approval from the Institutional Review Board (approval no.: 1510) and adhered to the principles outlined in the Declaration of Helsinki. Informed consent was not required due to the retrospective design of the study and the utilization of deidentified clinical and institutional data.

### Participants

Inclusion criteria were patients with a diagnosis of osteoarthritis (OA), undergoing primary cemented TKA and with complete blood type information available. The following exclusion criteria were applied to remove patients from the study: (1) patients diagnosed with rheumatoid arthritis, knee osteonecrosis, Charcot joint, or bleeding disorders (e.g., hemophilia, von Willebrand disease); (2) patients with a history of prior surgical intervention on the affected knee; (3) cases in which a prosthetic insert other than the cruciate-retaining (CR) type was used; (4) patients who had been receiving anticoagulant or antiplatelet therapy within 2 weeks before surgery; and (5) patients with major systemic comorbidities known to affect perioperative bleeding risk, such as chronic liver disease, end-stage renal disease, or hematologic malignancies. In addition, subjects lacking sufficient or complete clinical data necessary for analysis were excluded from the study.

### Data collection

Relevant patient data were retrospectively extracted from the patients’ electronic health records.

### Preoperative factors

Preoperative factors were collected to assess homogeneity between the four blood type groups. Baseline demographics included: age; sex; blood type (A, B, AB, or O); height; body weight; body mass index (BMI); preoperative hemoglobin (Hb) (g/dL); preoperative hematocrit (Ht) (%); preoperative international normalized ratio (INR); preoperative platelet count (104/μL); preoperative prothrombin time (PT) (%); preoperative activated partial thromboplastin time (APTT) (s).

### Intra- and postoperative factors

All procedures were performed by three experienced orthopedic surgeons, all of whom regularly perform total knee arthroplasty. A supervising surgeon, who was one of the three, was present for all operations to ensure consistency. The surgical technique was standardized and included the use of a medial parapatellar approach, a cemented cruciate-retaining prosthesis, and an air tourniquet. The tourniquet was inflated immediately before the skin incision and deflated just after wound closure. No drains were used in any patient. Intraoperative and postoperative management were also standardized. All patients received an intraarticular injection of tranexamic acid (500 mg) during surgery. Thromboprophylaxis included mechanical measures (compression stockings and sequential pneumatic pumps) and pharmacological prophylaxis using edoxaban (15 mg orally for 2 weeks starting the day after surgery). Postoperative rehabilitation was initiated on postoperative day 1 according to the institutional protocol. Transfusion was considered when hemoglobin level dropped below 7.0 g/dL, or below 8.0 g/dL in patients with symptoms or underlying cardiopulmonary conditions. Intra- and postoperative variables were collected as follows: surgical time (min); whether blood transfusion was performed; postoperative hemoglobin (g/dL); postoperative hematocrit (%); and postoperative D-dimer (μg/mL). At the study institution, it is standard practice to obtain a blood sample on postoperative day 7 for all patients.

### Calculation of blood loss

Total blood loss was estimated using the Gross formula [18]: total blood loss (mL) = blood volume (mL) × (preoperative Ht (%) − postoperative Ht (%)) / mean Ht (%), where blood volume was calculated using the Nadler formula on the basis of the patient’s age, weight, and height: blood volume (male) = 604 + 0.0003668 × [height (cm)]3 + 32.2 × weight (kg), blood volume (female) = 183 + 0.000356 × [height (cm)]3 + 33 × weight (kg) [19].

### Statistical analysis

Continuous values were expressed as the mean and standard deviation (SD). Participants were categorized into four cohorts on the basis of ABO blood group: A, B, O, and AB. All statistical analyses were conducted using PASW Statistics 25 (SPSS Inc., Chicago, IL, USA). Depending on the data distribution, one-way analysis of variance (ANOVA) followed by Tukey’s post hoc test or chi-squared testing was employed to assess group differences. To compare outcomes between blood type O and non-O groups, we first calculated propensity scores using a multivariable logistic regression model with age, sex, BMI, and operative time (defined as the duration from immediately before skin incision to immediately after wound closure) as covariates. IPTW was then applied on the basis of these propensity scores to adjust for baseline imbalances. After weighting, the standardized mean differences (SMDs) for these variables were calculated to assess covariate balance. Subsequently, the Mann–Whitney *U* test was applied to compare outcomes between the weighted groups. As this study was exploratory in nature, no a priori power analysis was conducted. A post hoc power analysis was performed using G*Power 3.1 software on the basis of the observed effect size (Cohen’s d = 0.522), an alpha level of 0.05, and a desired power of 0.95. This analysis was conducted for exploratory purposes only. Statistical significance was set at *P* < 0.05 for all analyses.

## Results

**Fig. 1:**
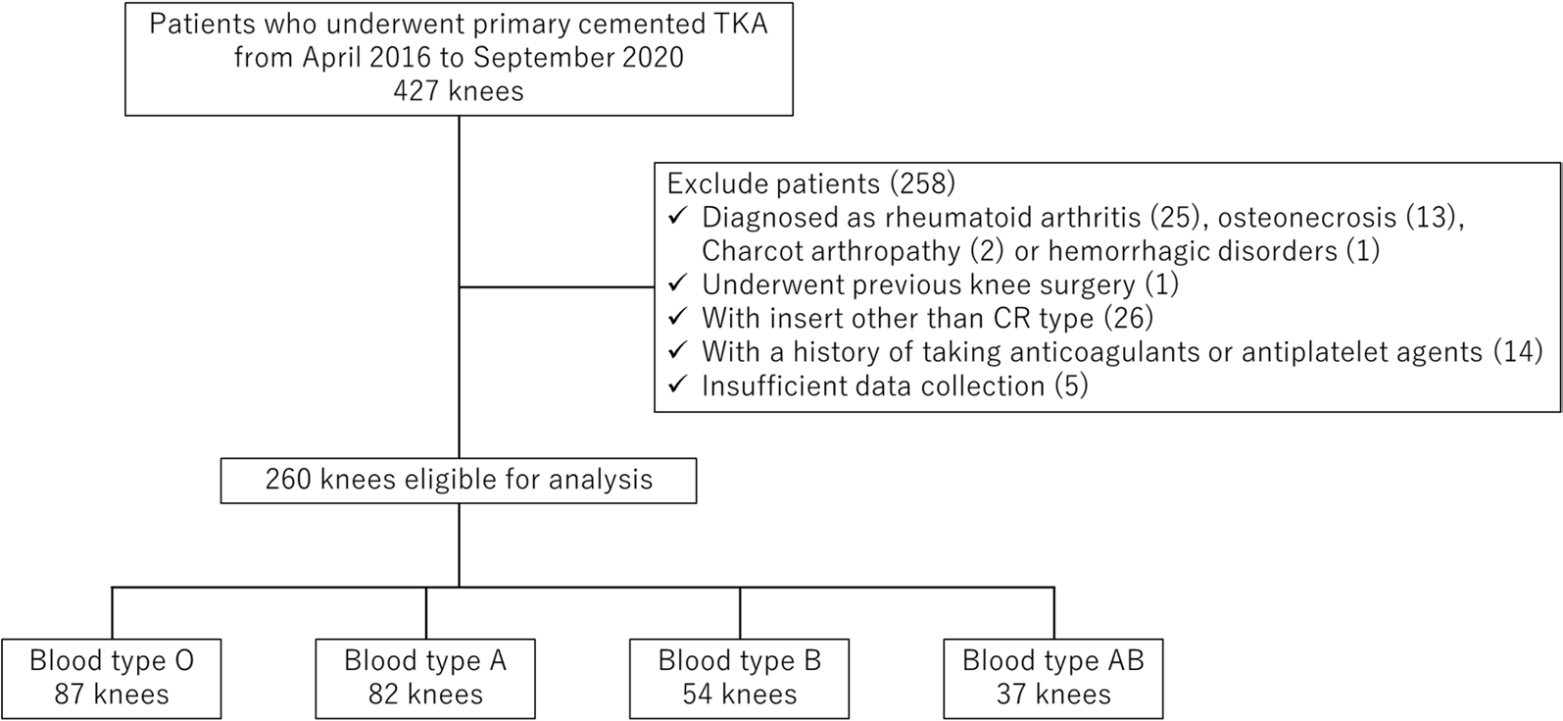
Flow diagram of patient selection. *TKA* total knee arthroplasty, *CR* cruciate-retaining

**Fig. 2:**
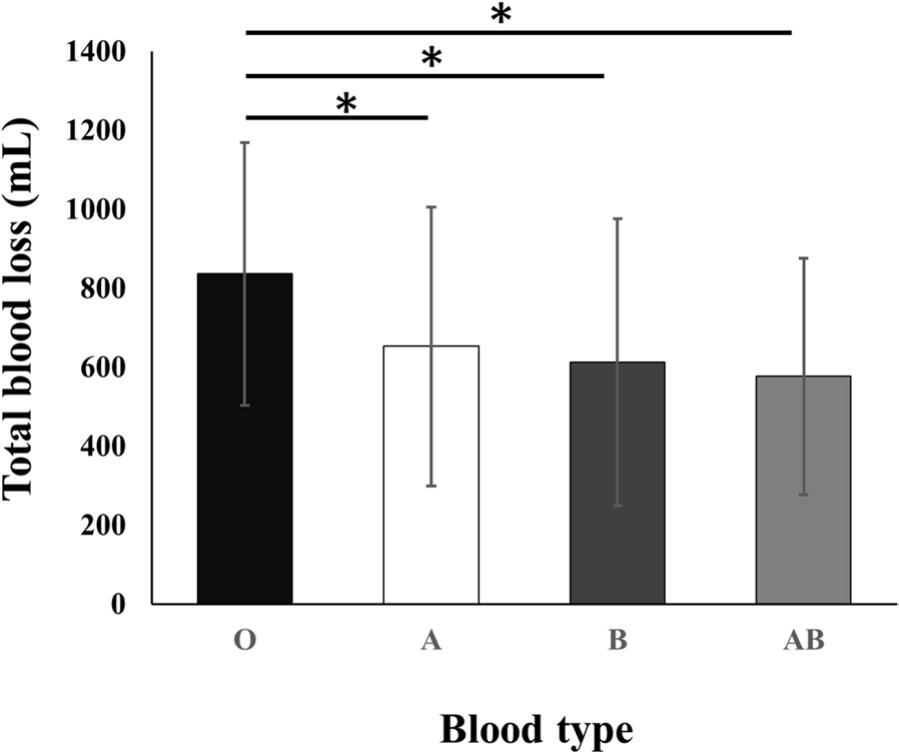
Comparison of total blood loss between blood type A, B, O and AB. Patients with blood Type O had significantly higher blood loss than all other blood types. *: P < 0.05

**Fig. 3:**
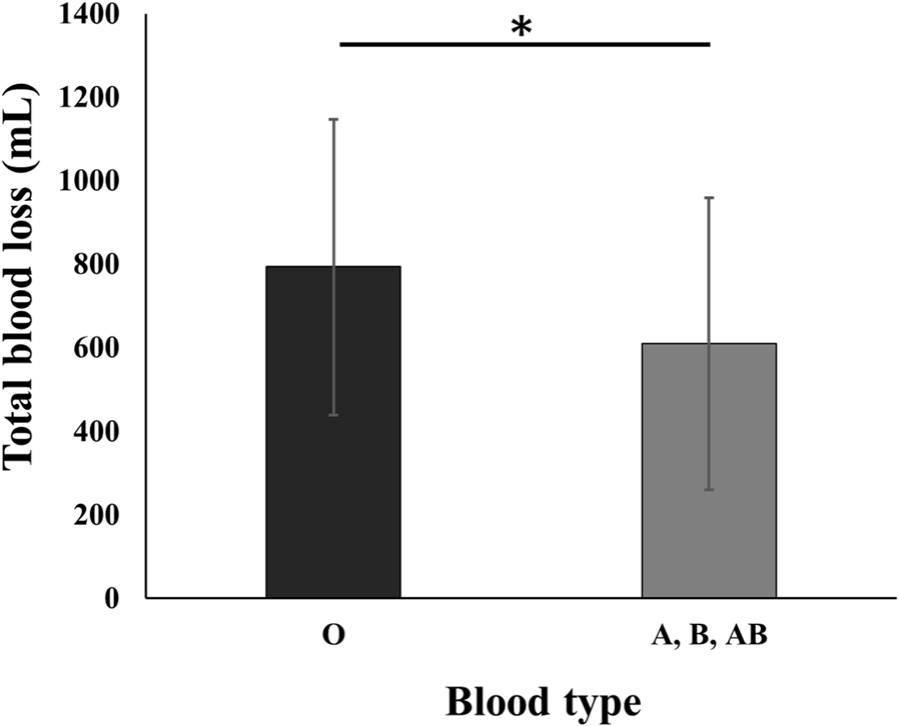
After adjustment for baseline characteristics using IPTW, comparison of total blood loss between blood type O and other blood types. Patients with blood type O had significantly higher blood loss than other blood types. *: *P* < 0.05

Of 427 potentially eligible knees, 260 met the inclusion criteria and were analyzed (Fig. 1). The participants were stratified into four groups according to their ABO blood type: type O, 87 (33%); type A, 82 (32%); type B, 54 (21%); and type AB, 37 (14%). Table 1 presents the baseline and perioperative characteristics, revealing no significant differences in age, sex, BMI, preoperative blood tests, blood volume or surgical time between groups. The mean total blood loss was 829 ± 370 ml for type O, 702 ± 384 ml for type A, 574 ± 361 ml for type B, and 528 ± 282 ml for type AB. Patients with blood type O exhibited significantly greater blood loss compared with those with other blood types. (Fig. 2). Table 2 summarizes the clinical characteristics following IPTW adjustment. After adjustment for baseline characteristics, the mean total blood loss was 794 ± 354 ml for type O and 610 ± 350 ml for the other blood types, with type O showing significantly higher blood loss than the other blood types (Fig. 3).

**Table 1:** Baseline characteristics and perioperative information in each blood types

	Type O, *n* = 87	Type A, *n* = 82	Type B, *n* = 54	Type AB, *n* = 37	*P*-value
Characteristics					
Age (years)	73.5 ± 9.2	71.1 ± 11.7	75.0 ± 9.2	72.6 ± 9.4	0.16
Sex (male/female)	21/66	20/62	12/42	7/30	0.52
BMI (kg/m^2^)	27.0 ± 5.2	26.8 ± 5.8	25.7 ± 3.3	23.5 ± 4.2	0.17
Preoperative blood test					
Hb (g/dL)	12.6 ± 1.5	12.7 ± 1.8	12.2 ± 1.6	12.0 ± 1.4	0.22
Ht (%)	38.4 ± 4.1	38.6 ± 5.0	37.3 ± 4.6	36.2 ± 3.3	0.07
INR (%)	1.01 ± 0.07	1.00 ± 0.07	1.00 ± 0.09	0.99 ± 0.09	0.54
Platelet (10^4^/μl)	24.2 ± 6.1	24.8 ± 7.2	22.7 ± 5.7	21.1 ± 5.6	0.06
PT (%)	98.5 ± 11.9	97.3 ± 17.1	101 ± 16.0	108 ± 21.2	0.53
APTT (seconds)	30.3 ± 3.8	29.5 ± 3.2	29.3 ± 3.1	29.8 ± 3.4	0.44
Perioperative information					
Surgical time (minutes)	108 ± 24.6	97.8 ± 24.4	86.8 ± 19.3	96.7 ± 23.1	0.22
Number of transfused (cases)	0	0	0	0	-
Blood volume (mL)	3732 ± 819	3669 ± 862	3579 ± 709	3228 ± 569	0.52
Postoperative blood test					
Hb (g/dL)	10.0 ± 1.3	10.4 ± 2.1	10.4 ± 1.4	10.1 ± 1.2	0.47
Ht (%)	30.9 ± 3.7	32.2 ± 4.3	31.5 ± 3.8	30.3 ± 3.0	0.13
D-dimer (μg/mL)	7.9 ± 4.1	9.1 ± 4.6	8.6 ± 5.1	9.9 ± 6.6	0.30

The data are given as mean ± standard deviation

*BMI* body mass index, *Hb* hemoglobin, *Ht* hematocrit, *INR* international normalized ratio, *PT* prothrombin time, *APTT* activated partial thromboplastin time

**Table 2:** The comparison of characteristics between blood type O and other blood types after adjustment using IPTW

	Type O n = 87	Non-O type n = 173	SMD
Characteristics			
Age (years)	73.1 ± 9.0	72.9 ± 10.5	0.024
Sex (male/female)	16.6/70.5	32.8/140.1	0.005
BMI (kg/m^2^)	26.2 ± 5.2	26.3 ± 5.3	0.016
Perioperative information			
Surgical time (minutes)	103 ± 24.9	100 ± 27.1	0.030

The data are given as mean ± Standard Deviation

*IPTW* inverse probability of treatment weighting, *BMI* body mass index, *SMD* standardized mean difference

Values represent weighted means and proportions after IPTW. Sample sizes (n) represent unweighted actual counts

## Discussion

The main finding of this study is that individuals with blood type O appear to be at increased risk of perioperative blood loss in TKA compared with other blood types, supporting our hypothesis. This is an important finding, given the lack of previous research on the relationship between blood type and postoperative blood loss in TKA. Although transfusion rates during TKA have declined in recent years [20], the aggregate number of transfusions may still rise. This is due to the rising number of primary TKA procedures, which totaled 480,958 cases in the USA in 2019 and is projected to reach 1,222,988 by 2040 and 2,917,959 by 2060 [21]. Given the reports of prolonged hospital stays in cases requiring blood transfusions, the study is also important from a health economic perspective [2, 3].

There are some previous studies investigating the association between blood type and perioperative blood loss in other surgical procedures; some studies have reported an increased risk of adverse outcomes for patients with blood type O in trauma and other critical care settings, while others have found no significant differences on the basis of blood type in extensive spine surgery [22]. In that report, although the patient background was matched precisely, due to the nature of major spinal surgery, it is expected that the variation in surgical invasion and blood loss during surgery will be greater from case to case compared with primary TKA, which may have led to the difference from the current study. A strength of the present study is the reduced case-specific variation in blood loss attributed to surgical technique due to the standardized nature of the primary TKA procedure. This uniformity minimizes case-specific differences in surgical invasiveness and allows for a more accurate comparison of blood loss between blood types. The underlying mechanisms responsible for the observed association between blood type O and increased perioperative blood loss during TKA remain unclear. One possible hypothesis is that blood type antigens may influence platelet adhesion and aggregation, which are critical processes in coagulation and fibrinolysis [23, 24]. Another hypothesis is that the lower plasma levels of vWF in type O individuals—reported to be 25–30% less than those in non-O individuals—may impair hemostatic function and enhance bleeding risk [25, 26]. However, vWF levels were not assessed in this study, and thus this explanation remains speculative. The discussion is based on prior literature and should be interpreted as a hypothesis rather than a data-driven conclusion. vWF is integral to primary hemostasis, facilitating platelet adhesion to injured endothelium and promoting platelet aggregation. It also stabilizes factor VIII, protecting it from premature degradation [27–29]. Consequently, the reduced vWF levels may partially account for the increased blood loss observed in type O patients. A recent meta-analysis corroborated these findings, showing that individuals with blood type O have a significantly higher incidence of bleeding complications [30]. Further investigation is required to elucidate these mechanisms and to determine the extent to which they contribute to the increased blood loss observed in patients with blood type O undergoing TKA.

This study has some limitations. One limitation is that Hb level was measured only on postoperative day 7, as per our institution’s routine protocol. Although previous studies have reported that Hb levels typically reach their lowest point around 3 days after TKA and begin to recover from postoperative day 4–5 [31], we were unable to capture such temporal changes. Therefore, caution is needed when interpreting the timing and extent of postoperative anemia on the basis of our dataset. In addition, while a post hoc power analysis was conducted to provide supplementary context regarding the observed effect size, we acknowledge that such analyses have inherent limitations due to their reliance on observed data, which may lead to circular reasoning. As a result, the post hoc power analysis should not be interpreted as confirmatory evidence of statistical robustness. Future prospective studies with a priori power calculations based on predefined effect sizes are needed to validate our findings.

The findings of this study have significant clinical implications. Although the mean difference in blood loss between type O and non-O groups was approximately 180 mL, which may appear modest, it could be clinically relevant in certain subgroups of patients, such as those with preoperative anemia or reduced cardiopulmonary reserve. Further prospective studies are needed to clarify the clinical implications of this finding. While this study quantified perioperative blood loss, it did not assess clinical outcomes such as transfusion rates, postoperative complications, or recovery measures. Therefore, the results should be interpreted as preliminary evidence of an association. Future studies should incorporate both surrogate and patient-centered outcomes to better determine the clinical impact of blood type on perioperative risk and recovery. Surgeons and anesthesiologists should be aware of the potential for increased perioperative blood loss in patients with blood type O undergoing TKA. Enhanced vigilance in monitoring blood loss, along with consideration of preemptive measures such as blood product transfusion or the use of targeted coagulation therapies, may be warranted for this patient group. Future research should aim to substantiate the observed correlation between blood type and perioperative blood loss in TKA in larger, more diverse cohorts. Additionally, studies focused on exploring the underlying biological mechanisms will be crucial in developing targeted interventions that could mitigate the increased bleeding risk in patients with blood type O.

This investigation offers novel perspectives on the association between ABO blood group and perioperative blood loss in TKA. The findings suggest that type O patients may be at greater susceptibility to blood loss in the perioperative phase of TKA, and further research is needed to investigate the underlying mechanisms and potential interventions. Clinicians should be aware of this potential risk in type O patients and monitor blood loss closely during and after TKA.

## Data Availability

The datasets generated and/or analyzed during the current study are available from the corresponding author on reasonable request.

## Abbreviations

APTT: Activated partial thromboplastin time
BMI: Body mass index
CR: Cruciate-retaining
Hb: Hemoglobin
Ht: Hematocrit
INR: International normalized ratio
IPTW: Inverse probability of treatment weighting
OA: Osteoarthritis
PT: Prothrombin time
SD: Standard deviation
TKA: Total knee arthroplasty
vWF: Von Willebrand Factor
